# Integration of Ego-States Therapy into a Day Clinic Approach for Addictive Disorders: A Case Report

**DOI:** 10.1192/j.eurpsy.2025.2137

**Published:** 2025-08-26

**Authors:** U. Altunoez

**Affiliations:** Department of General Psychiatry and Psychotherapy, KRH Wunstorf Psychiatric Hospital, Wunstorf, Germany

## Abstract

**Introduction:**

The relationship between childhood traumatic experiences and substance use disorders (SUD) has been the subject of considerable discussion in the literature. The development of efficacious therapeutic models for the treatment of these comorbidities remains an ongoing area of research. In particular, trauma-related disorders accompanied by dissociative changes and SUD may be effectively treated with ego-states therapy, which employs a combination of psychoanalytic and hypnoanalytic methods with other systemic approaches.

**Objectives:**

The objective of this presentation is to illustrate a case study from an interdisciplinary day clinic that employs an integrated approach to the treatment of substance use disorders (SUD) and other comorbidities. Furthermore, we will propose an innovative naturalistic method for addressing complex phenomenological manifestations of trauma-related disorders in individuals with SUD.

**Methods:**

In this presentation, we will introduce a psychotherapeutic method from a day clinic in Lower Saxony, Germany, through a comprehensive case study.

**Results:**

Case:

A 40-year-old woman was referred to the day clinic following a period of ward treatment following the death of her father. This period led to an increase in her cannabis consumption and a concomitant increase in depressive symptoms. In her history, she revealed that her elder sister had committed suicide when the patient was in primary school. Consequently, she was tasked as a child with the responsibility of handling all bureaucratic matters with her parents, as she was the only member of her family who could speak German due to their migration background.

During the psychotherapeutic treatment, various ego states were identified and addressed through a combination of psychoanalytic, systemic-milieu therapeutic, and family therapeutic interventions. Particular attention was paid to those aspects that had been functional during childhood but were now manifesting in a destructive manner. The adult ego-states were activated to bring those processes into conscious awareness and promote psychological stabilization. By the end of the 6-week day clinic process, cannabis levels were no longer detected in the urine, and a significant reduction in depressive symptoms was observed.

**Conclusions:**

Complex phenomenological presentations require multidisciplinary, holistic, and integrative therapeutic approaches. Further evidence-based evaluations are necessary to optimize treatment outcomes for such cases.

**Disclosure of Interest:**

None Declared

